# Late Diagnosis of a Patient With Gradual Onset of Lateral Medullary Syndrome Secondary to Spontaneous Vertebral Artery Dissection: A Case Report

**DOI:** 10.7759/cureus.24934

**Published:** 2022-05-12

**Authors:** Dunya Alfaraj, Mohammed A Alhamoud, Faleh M Alotaibi

**Affiliations:** 1 Emergency Department, King Fahd University Hospital, Imam Abdulrahman Bin Faisal University, Dammam, SAU; 2 Emergency Medicine, College of Medicine, Imam Abdulrahman Bin Faisal University, Dammam, SAU; 3 Emergency Medicine, King Fahd University Hospital, Dammam, SAU

**Keywords:** stroke, posterior circulation stroke, vertebral artery thrombosis, lateral medullary syndrome (wallenberg syndrome), vertebral artery dissection

## Abstract

Lateral medullary syndrome (LMS), also known as Wallenberg syndrome, is a cerebrovascular event following ischemia of the lateral part of the medulla oblongata. Some of its etiologies include atherosclerotic changes, hypertension, thromboembolism, vertebral artery dissection (VAD), and aneurysm. We present a case of a 45-year-old male with LMS with a gradual onset of atypical symptoms of LMS, which has led to a late diagnosis of our patient. VAD is a commonly recognized cause of stroke in young people and it is a more frequent cause of LMS than posterior inferior cerebellar artery diseases. This case highlights the importance of early identification of signs and symptoms and that appropriate investigation should take place to optimize patient life quality and prognosis.

## Introduction

Lateral medullary syndrome (LMS), also known as Wallenberg syndrome, is a cerebrovascular event following ischemia of the lateral part of the medulla oblongata. The vertebral artery and the posterior inferior cerebellar artery are the frequently involved arteries in the development of this syndrome [1,2]. Precipitant factors of LMS include thromboembolic events caused by atherosclerosis, hypertension, and vertebral artery diseases, such as dissection, aneurysm, and hypoplasia. Although hypoplasia alone is not a leading cause of LMS, but if present with other risk factors, it might lead to LMS [1,3,4].

We present a case of a 45-year-old male with LMS with a gradual onset, lacking some of the typically seen symptoms that are reported to be common among patients with LMS, which has led to early misdiagnosis of our patient's condition.

## Case presentation

**Figure 1:**
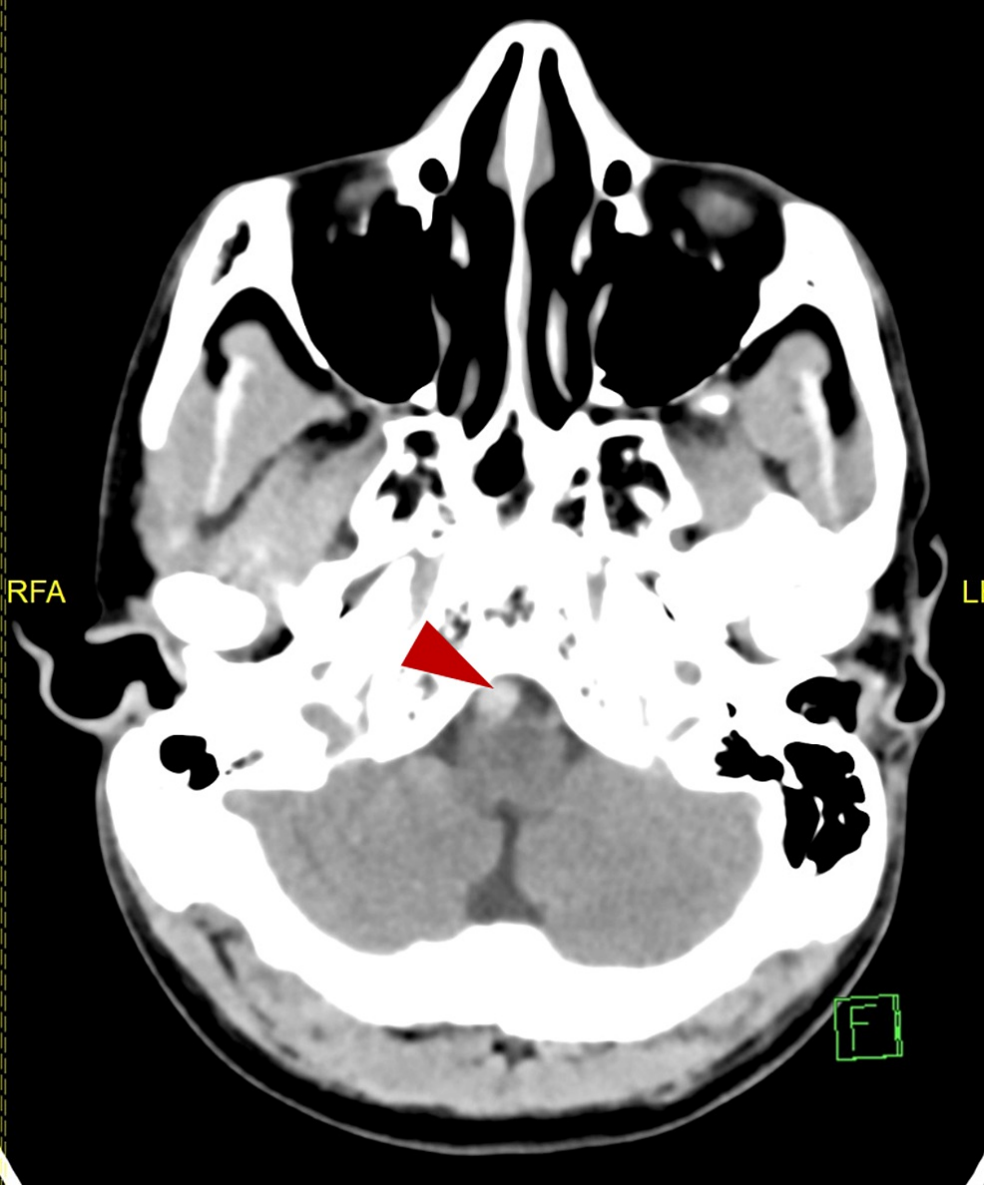
Computed tomography. Arrowhead showing hyperdensity of the right vertebral artery.

A 45-year-old Indian non-smoker male, who was not known to have any medical illnesses, presented to the emergency department of a tertiary hospital complaining of headache associated with recurrent episodes of vomiting for five days. On examination, he looked drowsy and diaphoretic, and his pupils were reactive bilaterally. His blood pressure was 247/130; therefore, the patient was given 40 mg of intravenous labetalol. Head CT was ordered to rule out intracranial insult. It showed hyperdensity of the right vertebral artery within the distal segment (Figure 1), which was attributed to atherosclerotic changes, with no other findings.

**Figure 2:**
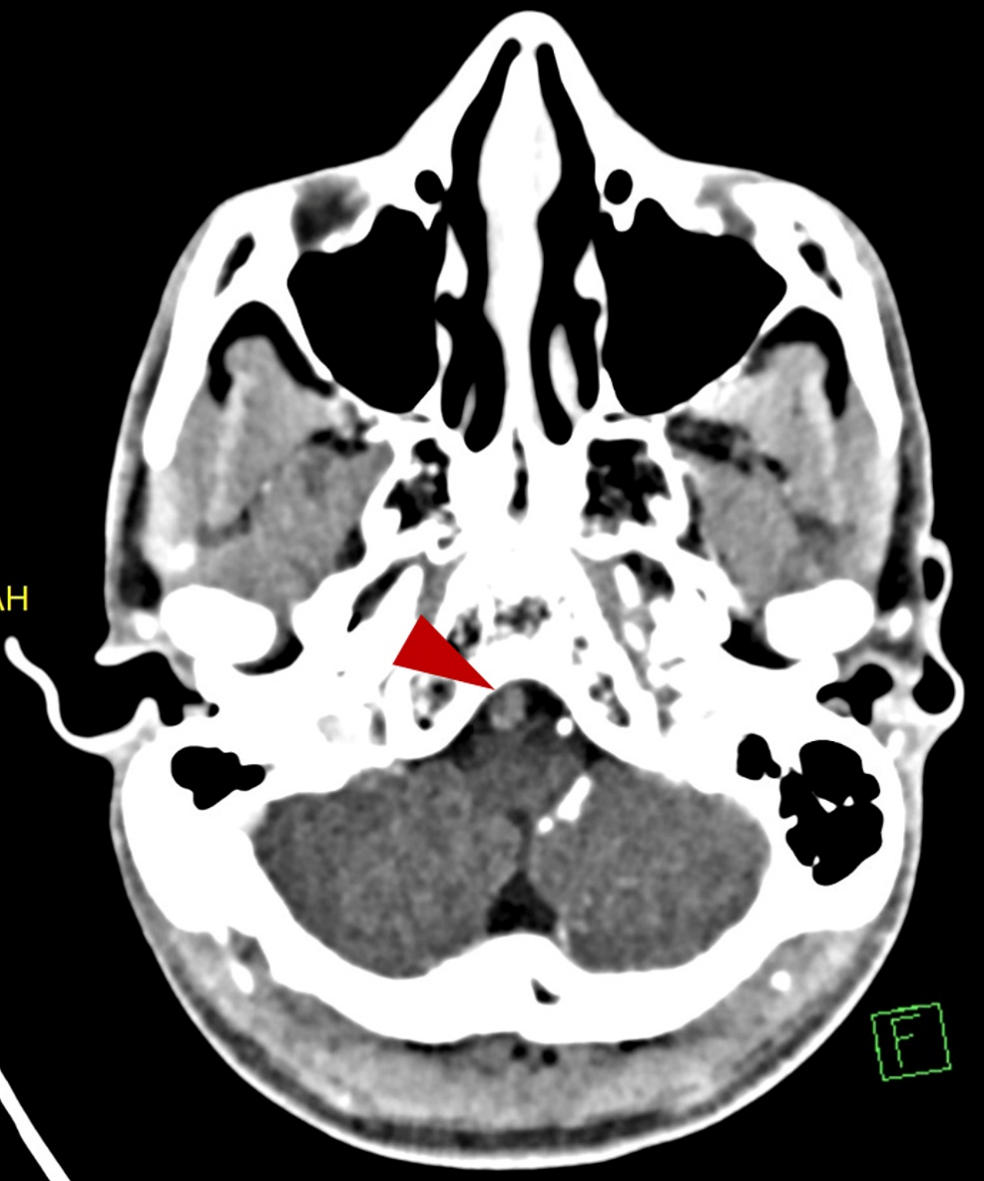
CT angiography. Arrowhead showing vertebral artery dissection with a thrombosed aneurysm.

After the administration of labetalol, the patient became stable, and his headache was relieved. Accordingly, he was diagnosed with hypertension; hence, he was instructed to follow up at a primary healthcare center and prescribed valsartan and hydrochlorothiazide, and discharged home. Ten hours later, he revisited the ED with a new complaint, which was hiccups, and he had a blood pressure of 180/100. On physical examination, there were no signs of lateralization, pupils were reactive, and he had a normal neurological exam. Metoclopramide and pantoprazole were administered, and the patient showed signs of improvement and thus was discharged. At night and after nine hours of discharge, the patient came back to the ED for the third time. Besides headache, vomiting, and hiccups, he reported new symptoms that developed suddenly, which were dysphagia, gait disturbance, and dizziness. He stated that his dysphagia was to both solids and liquids and that he involuntarily expelled everything he consumed. As for the headache, it was in the occipital area, used to increase in intensity during the morning, and it was 7/10 in severity. There was no history of change in the level of consciousness, weakness, numbness, or neck stiffness. On examination, the patient appeared alert and oriented to time, place, and person. His pupils were reactive to light bilaterally, and extraocular movements, visual field, and acuity were intact. There was nystagmus to the left side, right-sided facial palsy, ataxic wide-based gait, uvula deviated to the left, and diminished gag reflex. There was no pronator drift with normal muscle tone, 5/5 power, and +2 reflexes overall. Light touch, vibration, proprioception, pinprick, and temperature sensations were normal. He also looked dehydrated. His blood pressure on the third visit was 188/118. The patient was diagnosed as a case of acute stroke; therefore, CT angiography (CTA) was ordered. CTA revealed dissection of the right vertebral artery with a thrombosed aneurysm (Figure 2).

He was admitted and given aspirin, atorvastatin, pantoprazole, metoclopramide for hiccups, enoxaparin sodium, and lisinopril. A nasogastric tube was inserted, and he was kept nothing per mouth (NPO) to prevent aspiration. The neurosurgery team was consulted about the possibility of intervention. A limited brain MRI study was done showing diffusion restriction at the right lateral medulla oblongata denoting acute/early subacute ischemic changes, and the patient was diagnosed with acute LMS with right vertebral artery dissection (VAD). The patient was subsequently admitted for further management.

## Discussion

VAD is a commonly recognized cause of stroke in young people [5]. VAD can be classified as either spontaneous or traumatic. Traumatic dissection may be sustained by either penetrating or blunt trauma, including excessive flexion or extension of the neck, motor-vehicle accidents, chiropractic neck manipulation, and assault [6-8]. Connective tissue diseases, such as Ehlers-Danlos syndrome and Marfan syndrome, are known to give rise to spontaneous VAD [9]. It is believed that vertebral artery diseases are more frequent causes of LMS than posterior inferior cerebellar artery diseases [10]. Ipsilateral Horner syndrome, limb ataxia, loss of pain and temperature sensation over half of the face, and contralateral hypoalgesia of the body are all sensitive findings of LMS. Other unspecific but frequent symptoms are vertigo, dizziness, nausea, vomiting, and headache [1,11,12]. Diversity of presentations occurs when areas in danger are supplied by collateral flow or through residual perfusion.

Our patient had dysphagia, hiccups, and uvula deviation to the left, which could be attributed to an affected nucleus ambiguous in the dorsolateral medullary oblongata. He also had vertigo, vomiting, and nystagmus, which can be indicative of pathology of the vestibular nuclei [1,2,10,13]. What makes our case atypical is that the patient had preserved crossed pain and temperature sensation over his ipsilateral half of the face and contralateral limb, which are controlled by the trigeminal nucleus and tract, and spinothalamic tract, respectively. In addition, he did not develop Horner’s syndrome, which is the result of affected descending sympathetic fibers [14].

Although it is relatively rare to be reported in patients with LMS, our patient had ipsilateral facial palsy, and a similar case was demonstrated with this manifestation [14]. The explanation of this could be due to the disruption of corticobulbar fibers that ascend in the dorsolateral medulla to the contralateral facial nucleus after they decussate [15,16]. The lack of some of the typical and sensitive findings in our case was the reason why a rapid clinical diagnosis was not made until the third visit to the emergency department. Assessing a suspected case of LMS should include complete history taking, thorough physical examination, and diagnostic studies. Risk factors of stroke should be evaluated, for instance, hypertension, smoking, and heart disease. Other history findings that may be indicative of VAD are trauma, headache, and neck pain [1,17]. Even though a head CT scan is the initially ordered imaging modality in suspected patients with LMS, early ischemic changes may be invisible in the first few hours [15]. This may explain why the first ordered head CT did not show ischemic changes. On the other hand, brain MRI offers better visualization of infarcted areas in the lateral medulla [6]. Heparin and intravenous tissue plasminogen activator should be considered in acutely presented patients with the consultation of the acute stroke care team if there are no contraindications of either statin or endovascular thrombectomy [1,18]. Patients with LMS have a good prognosis, and most of them recover their abilities to proceed with their life activities within one year, although approximately 10% of patients die in the initial presentation [2,14,18].

They need physical therapy to help them restore their strength, and stroke prevention must be taken into consideration by modifying and controlling risk factors such as hypertension and smoking. Patients with dysphagia may be considered for gastronomy tube placement to help them nourish and minimize the risk of aspiration until they regain their swallowing ability. Patients should also be followed up by speech therapists for rehabilitation, which is essential for their daily self-care activities [1].

## Conclusions

LMS remains a common cause of stroke among young people with a wide variety of presentations. It has favorable predicted outcomes with improved care and rehabilitation. Early identification of signs and symptoms, with complete neurological physical examination and appropriate investigations, should take place to optimize patient life quality and prognosis.
